# The association of physical activity and cholesterol concentrations across different combinations of central adiposity and body mass index

**DOI:** 10.15171/hpp.2016.21

**Published:** 2016-08-10

**Authors:** Paul D. Loprinzi, Ovuokerie Addoh

**Affiliations:** ^1^Director of Research Engagement – Jackson Heart Study Vanguard Center of Oxford, Physical Activity Epidemiology Laboratory, Department of Health, Exercise Science and Recreation Management, The University of Mississippi, University, MS 38677, USA; ^2^Physical Activity Epidemiology Laboratory, Department of Health, Exercise Science and Recreation Management, The University of Mississippi, University, MS 38677, USA

**Keywords:** Body mass index, Epidemiology, Exercise, NHANES, Waist circumference

## Abstract

**Background:** The purpose of this study was to investigate if those who are physically active,compared to physically inactive, have better cholesterol profiles across different combinations of body mass index (BMI) and waist circumference (WC).

**Methods:** Data from the 1999-2006 National Health and Nutrition Examination Survey (NHANES) were used (N = 16 095). Cholesterol parameters included total cholesterol (TC), high density lipoprotein cholesterol (HDL-C), TC/HDL-C ratio, triglycerides and at herogenic index(Log10 [triglycerides/HDL-C]). Physical activity (PA) was assessed via self-report, with BMI and WC objectively measured. Cholesterol concentrations of 6 combinations of BMI and WC were evaluated among active and inactive participants. Multivariable linear regression analysis was utilized.

**Results:** Findings were not consistent across sex. There was little evidence to suggest an association of PA on TC across varying BMI and WC combinations. For example, among those who had an obese BMI and high WC, inactive participants did not have different TC level when compared to active participants (β = -1.2; 95% CI: -3.9-1.5, P = 0.38). There was evidence to suggest a favorable association of PA on HDL-C, triglycerides and at herogenic index across varying BMI and WC combinations. For example, among those who had an obese BMI and high WC, inactive (vs. active) participants had a lower HDL-C (β_adjusted_ = -1.6, P < 0.01). When considering either gender, there was sufficient evidence to suggest a favorable association of PA on at least one of the evaluated cholesterol parameters for each of the BMI/WC combinations with the exception of normal BMI and high WC.

**Conclusion:** Except for those having normal weight central obesity, PA is favorably associated with cholesterol parameters across various combinations of BMI and WC.

## Introduction

Obesity is an important risk factor for various chronic diseases, such as diabetes, hypertension, dyslipidemia, coronary heart disease and mortality.^1^ Due to the convenience of measuring body mass index (BMI), coupled with epidemiological findings that BMI is associated with morbidity and mortality,^1^ BMI is a clinical standard for the identification of patients at risk for adiposity-related morbidities.

In addition to the important clinical implications of BMI assessment, body fat distribution is also an important risk factor for various obesity-related chronic diseases. As such, waist circumference (WC) is an often considered surrogate marker of central fat mass^2^ and it associates with morbidity and mortality to a similar extent to waist-to-hip ratio (see Table 4.1 in the WHO expert consultation^3^). In fact, WC, even across various ranges of BMI, spanning from normal BMI to obese BMI, predicts worse health outcomes, such as early mortality^4^ and dyslipidemia.^5^ Further, both WC and BMI are independently associated with dyslipidemia,^5,6^ underscoring the potential clinical importance of considering both BMI and WC.

In addition to the observed independent associations of BMI and WC on health,^5,6^ emerging work suggests that varied combinations of BMI and WC may have differential effects on a patient’s health.^7^ For example, Sharma et al^7^ demonstrated that patients with normal weight central obesity had the highest mortality risk when compared to other BMI and WC combinations. Considerations of characteristics that influence cholesterol concentrations among patients with varied BMI and WC combinations is important as cholesterol profile is a well-established indicator of chronic disease and early mortality.^8,9^

One such health characteristic to consider is regular participation in physical activity (PA). Research demonstrates that regular participation in PA is inversely associated with BMI, WC and cholesterol.^10^ However, the combined associations of BMI, WC and PA on dyslipidemia, is less clear. Thus, the purpose of this study was to address this PA-related obesity risk classification model (i.e., combinations of PA, BMI and WC) as it relates to patient cholesterol concentrations among a national sample (National Health and Nutrition Examination Survey, NHANES) of adults in the United States.

## Materials and Methods

### Study design 

The NHANES is an ongoing survey conducted by the Center for Disease Control and Prevention (CDC) designed to evaluate the health status of US adults through a complex, multistage, stratified clustered probability design. Participants are interviewed in their homes and then subsequently examined in a mobile examination center (MEC). Further information on NHANES methodology and data collection is available on the NHANES website (http://www.cdc.gov/nchs/nhanes.htm). Procedures were approved by the National Center for Health Statistics review board. Consent was obtained from all participants prior to data collection.

In the 1999-2000, 2001-2002, 2003-2004, and 2005-2006 cycles, the respective NHANES response rates for adults (20+ years) in the MEC were 68.3%, 71.6%, 68.1%, and 69.8%. These response rates are relatively high for epidemiological studies.

Participant data from the 1999-2006 NHANES were utilized. Analyses are based on data from 16 095 adults (20-85 years) who provided complete data for the study variables, inclusive of PA, BMI, WC, cholesterol, age, gender, race-ethnicity, cholesterol medication use, mean arterial pressure, C-reactive protein (CRP), previous year changes in PA, congestive heart failure, coronary artery disease, heart attack, emphysema, chronic bronchitis, stroke, diabetes and smoking status; a flow description of the participant sample size after exclusions is described in the results section.

In the 1999-2006 NHANES, 20 311 adults 20-85 years were enrolled. Among these, 2472 had missing PA, BMI or WC data (N_resultant_=17 839), as not all of the enrolled participants completed the examinations in the MEC. Among these 17839 participants, a further 292 were excluded because of having a BMI <18.5 kg/m^2^ (N_resultant_=17 547). Among these, a further 1452 participants were excluded because of missing data on the cholesterol concentrations or the covariates (N_resultant_=16 095). These 16 095 adult participants constituted the analytic sample.

### Obesity risk classification model

BMI was calculated from measured mass in kg divided by squared height in meters. Weight status was defined as BMI-determined normal weight (18.5-24.9 kg/m^2^), overweight (25-29.9 kg/m^2^) or obese (30+ kg/m^2^).^11,12^ WC was measured with a tape measure at the (uppermost) lateral border of the hip crest (ilium bone).^13,14^ High WC was defined as ≥102 cm for men and ≥88 cm for women.^15,16^ Consistent with current US government PA guidelines,^17^ participants were defined as “active” based on at least 2000 moderate-to-vigorous PA (MVPA) MET (metabolic equivalent of task)-min-month (assessment of MVPA described below).

Based on these three parameters (BMI, WC and PA), participants were classified into 6 mutually exclusive combinations of BMI and WC (normal BMI and normal WC; normal BMI and high WC; overweight BMI and normal WC; overweight BMI and high WC; obese BMI and normal WC; and obese BMI and high WC) among active and inactive participants (see Tables 1 and 2).

### Cholesterol assessment

High-density lipoprotein cholesterol (HDL-C), triglycerides, and total cholesterol (TC) were assessed enzymatically in serum or plasma via a blood sample. Notably, triglyceride data was only evaluated among ½ of the NHANES sample. The Hitachi 704 analyzer was used to calculate these cholesterol parameters. Specific details on the assessment of these cholesterol parameters is discussed thoroughly in a previous publication.^18^

### Physical activity

As described elsewhere,^19^ participants were asked open-ended questions about participation in leisure-time PA over the past 30 days. Data were coded into 48 activities, including 16 sports-related activities, 14 exercise-related activities, and 18 recreational-related activities.

For each of the 48 activities where participants reported moderate or vigorous-intensity for the respective activity, they were asked to report the number of times they engaged in that activity over the past 30 days and the average duration they engaged in that activity. For each activity, MET-min-month was calculated by multiplying the number of days, by the mean duration, by the respective MET level (MET-min-month = days*duration*MET level). The MET levels for each activity are provided elsewhere.^20^ As described elsewhere, this PA assessment has demonstrated evidence of convergent validity by positively associating with accelerometer-assessed PA.^19^

### Statistical analysis and covariates

Statistical analyses were performed via procedures from survey data using Stata (v.12; Stata Corp, College Station, TX). Due to multiple comparisons, statistical significance was set at *P*≤0.01. Analyses accounted for the complex survey design employed in NHANES by utilizing sample weights, primary sampling units and strata via the Taylor series (linearization) method. Sample weights were re-weighted to account for the use of combined NHANES cycles.^21^ Information on the use of sample weights to generate population weighted estimates is available elsewhere.^22^

Linear regression analysis was used to examine cholesterol (TC, HDL-C, TC/HDL-C and triglyceride) differences across the respective activity status groups (i.e., inactive vs. active across the different BMI/WC combinations), with results stratified by gender. For the multivariable linear regression models, covariates included *cholesterol medication use* (yes/no), *age* (years; continuous), *gender* (male/female)*, race-ethnicity* (Mexican American, non-Hispanic white, non-Hispanic black, and other), *mean arterial pressure* (mm Hg; continuous), *CRP* (mg/dL; continuous), *previous year changes in PA* (categorical; activity increased, decreased or stayed the same), *congestive heart failure* (yes/no), *coronary artery disease* (yes/no), *heart attack* (yes/no), *emphysema* (yes/no), *chronic bronchitis* (yes/no), *stroke* (yes/no), *diabetes* (yes/no) and *smoking status* (categorical; smokes every day, smokes some days, former smoker, never smoker). These covariates were selected based on previous research demonstrating their association with obesity, PA and cholesterol.^23^ Notably, results were similar when including all of these covariates versus a minimally adjusted model that did not include certain covariates (e.g., CRP) that may be involved in the mechanistic pathway between PA, obesity and cholesterol. Thus, each of these covariates were included in the adjusted models.

Age, gender, race-ethnicity, changes in PA, and smoking status were assessed via a self-report questionnaire. With regard to changes in PA, participants were asked, “*How does the amount of activity that you reported for the past 30 days compare with your PA for the past 12 months*? (Response options: more active, less active or about the same”.) Regarding the chronic diseases (congestive heart failure, coronary artery disease, heart attack, emphysema, chronic bronchitis, stroke, and diabetes), participants were asked if they had ever been told by a physician or other health professional that they had this disease. In addition to diabetes being assessed via self-report of physician diagnosis, here we also defined individuals as having diabetes if they had a fasting plasma glucose ≥126 mg/dL or an A1C ≥6.5%. Using the average of up to four manually assessed blood pressure measurements, mean arterial pressure was calculated using the following formula: ([(diastolic blood pressure × 2) + systolic blood pressure]/3). Lastly, high sensitivity CRP concentration was quantified using latex-enhanced nephelometry. Further details on the assessment of these laboratory-based parameters can be found elsewhere.^18^

## Results

Table 1 displays the study variable characteristics among these 16 095 analyzed participants, with results stratified by the obesity risk classification groups. Across these 12 groups, the sample size ranged from 90 participants (Group 9; obese BMI, normal WC and active) to 3442 participants (Group 12; obese BMI, high WC and inactive). Generally, those with a high WC were older than those with a normal WC. Women, compared to men, were more likely to have a high WC with a normal BMI. The active group compared to the inactive group, across all combinations of BMI and WC, generally had more favorable levels for all chronic diseases and biomarkers.

Table 2 displays the weighted regression associations (β, *P* value) examining the inactive vs. active groups for each evaluated cholesterol concentrations, with results presented for the entire sample and stratified by sex. Generally, unadjusted and adjusted results were similar. Across all BMI and WC combinations, there was no statistically significant difference in TC levels across activity status, with the exception of inactive women classified as obese BMI and normal WC having higher TC (35.5 mg/dL higher) than their active counterparts. With regard to HDL-C, those who were inactive (vs. active) had lower levels of HDL-C for all the BMI and WC combinations with the exception of normal BMI and high WC and obese BMI and normal WC. Results, however, were not consistent across sex, and results were similar for TC/HDL-C. For triglycerides, higher PA (vs. not meeting guidelines) was only associated with lower levels of triglycerides among three BMI/WC combinations: normal BMI and WC; overweight BMI and high WC; and obese BMI and high WC. For the atherogenic index, lower PA (vs. higher PA) was associated with a higher atherogenic index score for men with a normal BMI and normal WC, for women with an overweight BMI and a high WC, and for both men and women with an obese BMI and high WC. Collectively, and when considering either sex, meeting PA guidelines (vs. not) was favorably associated with at least one of the evaluated cholesterol concentrations for each of the BMI/WC combinations with the exception of normal BMI and high WC.

**Table 1:** Characteristics of the study variables (mean [95% CI]), 1999-2006 NHANES (N= 16095)^a^

**Variable**	**Normal BMI and** **normal WC**		**Normal BMI and High WC**		**Overweight BMI and** **normal WC**		**Overweight BMI and** **high WC**		**Obese BMI and** **normal WC**		**Obese BMI and** **high WC**	
**Group number**	**1**	**2**	**3**	**4**	**5**	**6**	**7**	**8**	**9**	**10**	**11**	**12**
	**Active**	**Inactive**	**Active**	**Inactive**	**Active**	**Inactive**	**Active**	**Inactive**	**Active**	**Inactive**	**Active**	**Inactive**
Sample size (n)	1971	2406	170	355	1237	1460	1102	2092	90	101	1669	3442
TC, mean mg/dL	191.8 (189-194)	195.5 (193-197)	210.7 (202-218)	211.1 (202-220)	200.2 (196-203)	204.9 (202-207)	207.7 (204-211)	213.1 (210-216)	201.2 (190-212)	200.2 (190-209)	204.4 (201-207)	205.5 (203-207)
HDL, mean mg/dL	60.4 (59-61)	57.7 (56-58)	60.6 (57-63)	58.8 (56-60)	49.9 (48-51)	48.9 (47-49)	54.7 (53-56)	52.2 (51-53)	45.8 (42-48)	47.2 (43-51)	47.2 (46-48)	47.4 (46-48)
Ratio of total/HDL	3.37 (3.3-3.4)	3.63 (3.5-3.7)	3.69 (3.4-3.9)	3.83 (3.5-4.0)	4.26 (4.1-4.3)	4.53 (4.4-4.6)	4.1 (3.9-4.2)	4.39 (4.3-4.5)	4.65 (4.2-5.0)	4.60 (4.1-5.0)	4.60 (4.4-4.7)	4.60 (4.5-4.7)
Triglyceride, mean mg/dL^b^	98.6 (95-102)	116.0 (109-122)	150.5 (130-171)	182.2 (103-261)	145.1 (132-157)	150.3 (139-161)	163.2 (135-191)	163.8 (155-171)	213.2 (112-313)	154.9 105204)	158.8(148-168)	181.2 (169-192)
Atherogenic index, mean mmol/L^c^	-.17 (-.19 to -.16)	-.11 (-.13 to -.08)	.009 (-.06-.08)	.03 (-.06-.13)	.03 (.004-.06)	.05 (.02-.09)	.03 (-.006-.06)	.09 (.07-.11)	.17 (.03-.31)	.03 (-.10-.16)	.10 (.07-.12)	.15 (.13-.17)
% Cholesterol medication	4.8	6.6	16.6	18.9	8.9	7.2	17.7	14.9	8.4	5.2	15.2	15.2
Age, mean yrs	40.8 (39-41)	44.3 (43-45)	54.6 (51-57)	56.5 (53-59)	41.7 (40-42)	43.4 (42-44)	51.1 (49-52)	53.0 (52-54)	36.1 33-38)	38.4 (35-41)	45.0 (43-46)	48.6 (47-49)
Male, %	45.7	46.5	7.9	8.4	80.0	78.2	40.4	35.1	87.6	83.7	53.5	38.5
Non-Hispanic white, %	78.9	69.5	85.6	79.3	74.1	58.7	84.0	72.6	53.9	40.6	73.8	69.9
Non-Hispanic black, %	6.8	10.1	3.4	6.3	9.0	12.6	5.1	8.7	18.9	24.0	12.0	13.7
Mexican American, %	4.3	8.6	2.9	4.6	7.0	14.3	4.7	8.2	10.0	23.7	5.5	8.5
MAP, mean mm Hg	85.2 (84-86)	86.0 (85-87)	88.1 (86-89)	88.5 (86-90)	88.0 (87-89)	89.0 (89-90)	89.3 (88-90)	90.1 (89-91)	89.6 (87-92)	88.9 (86-91)	90.8 (90-92)	91.4 (90-92)
CRP, mean mg/dL	0.21 (.19-.24)	0.30 (.26-.33)	0.28 (.21-.34)	0.38 (.32-.44)	0.27 (.22-.32)	0.36 (.29-.43)	0.38 (.33-.44)	0.47 (.44-.50)	0.29 (.22-.35)	0.40 (.15-.64)	0.51 (.48-.54)	0.70 (.66-.74)
MET-min-month, mean	10295.2	341.3	9402.2	260.1	10889.1	386.9	8326.5	329.8	12681.8	259.8	9079.1	327.7
Become less active in past year, %	18.0	22.2	20.9	28.7	16.8	26.3	15.3	24.7	19.1	20.1	17.5	24.8
Waist circumference, mean cm	81.1	82.3	92.2	92.5	92.6	93.0	99.5	99.9	97.1	96.9	112.4	113.7
BMI, mean kg/m^2^	22.3	22.3	23.9	23.6	26.9	26.8	27.8	27.8	31.2	30.9	34.8	35.7
CHF, %	0.6	1.8	0.2	3.0	0.7	1.7	1.7	4.2	0	1.4	1.4	3.8
CAD, %	1.2	2.7	3.4	4.0	2.9	1.9	6.4	4.7	0	2.8	3.5	4.5
Heart attack, %	1.1	3.2	2.3	3.9	2.3	2.7	4.2	5.1	0	4.5	3.0	4.8
Stroke, %	0.8	1.7	4.3	2.8	0.4	1.3	3.0	4.1	0	0.3	1.7	3.8
Emphysema, %	1.0	2.3	1.4	3.5	0.5	1.2	0.7	3.0	0	0	0.7	2.0
Chronic bronchitis, %	4.0	6.4	9.2	11.6	2.7	4.3	5.7	7.7	5.1	3.7	7.6	10.1
Diabetes, %	2.3	4.3	11.3	12.3	3.7	6.4	9.3	12.2	4.2	9.5	12.9	17.8
Daily smoker, %	17.6	33.6	13.9	25.7	14.9	22.7	13.7	21.3	19.9	23.8	15.3	19.0

Abbreviations: BMI, body mass index; CAD, coronary artery disease; CHF, congestive heart failure; CRP, C-reactive protein; HDL, high-density lipoprotein cholesterol; MAP, mean arterial pressure; MET, metabolic equivalent of task; WC, waist circumference. ^a^For categorical variables, percentages are reported. For continuous variables, means (95% CI) are reported. Normal BMI (18.5-24.9 kg/m^2^); Overweight BMI (25.0-29.9 kg/m^2^); Obese BMI (30+ kg/m^2^); Normal Waist Circumference (<102 cm for men and <88 cm for women); High Waist Circumference (≥102 cm for men and ≥88 cm for women); Active (≥ 2000 MET-min-month); Inactive (<2000 MET-min-month). ^b^ Triglyceride levels were only assessed among a ½ subsample of NHANES participants. In these analyses, 7899 participants had triglyceride data. The sample sizes across the 12 respective groups were: 966, 1211, 86, 176, 589, 701, 551, 1039, 46, 45, 816, and 1673. ^c^ Atherogenic index (expressed in mmol/L) calculated as follows: (log_10_(triglycerides/HDL-C). To convert HDL-C from mg/dL to mmol/L, divide by 38.67; to convert triglycerides from mg/dL to mmol/L, divide by 88.57.

**Table 2:** Weighted regression sex-specific associations (β, p-value) examining the inactive vs. active groups for each evaluated cholesterol parameters, 1999-2006 NHANES (N=16095)^a^

**Variable**	**Normal BMI and normal WC**		**Normal BMI and high WC**		**Overweight BMI and normal WC**		**Overweight BMI and high WC**		**Obese BMI and normal WC**		**Obese BMI and high WC**	
**Group Number**	**1**	**2**	**3**	**4**	**5**	**6**	**7**	**8**	**9**	**10**	**11**	**12**
	**Active**	**Inactive**	**Active**	**Inactive**	**Active**	**Inactive**	**Active**	**Inactive**	**Active**	**Inactive**	**Active**	**Inactive**
**TC**												
Entire Sample												
Unadjusted	Referent	3.7 (P=0.01)	Referent	0.33 (P=0.95)	Referent	4.7 (P=0.02)	Referent	5.4 (P=0.01)	Referent	-1.1 (P=0.86)	Referent	1.0 (P=0.48)
Adjusted^b^	Referent	1.9 (P=0.17)	Referent	-0.63 (P=0.92)	Referent	2.9 (P=0.15)	Referent	3.3 (P=0.11)	Referent	-1.2 (P=0.84)	Referent	-1.2 (P=0.38)
Men												
Unadjusted	Referent	4.9 (P=0.03)	Referent	-9.8 (P=0.65)	Referent	5.0 (P=0.03)	Referent	3.7 (P=0.25)	Referent	-6.1 (P=0.40)	Referent	-0.65 (P=0.76)
Adjusted^b^	Referent	3.3 (P=0.14)	Referent	-18.7 (P=0.41)	Referent	3.2 (P=0.16)	Referent	1.5 (P=0.65)	Referent	-7.2 (P=0.31)	Referent	-1.2 (P=0.57)
Women												
Unadjusted	Referent	2.8 (P=0.06)	Referent	1.3 (P=0.83)	Referent	4.4 (P=0.26)	Referent	6.0 (P=0.01)	Referent	29.9 (P=0.05)	Referent	1.6 (P=0.36)
Adjusted^b^	Referent	0.8 (P=0.55)	Referent	0.7 (P=0.90)	Referent	2.1 (P=0.56)	Referent	3.5 (P=0.13)	Referent	**35.5 (P=0.01)**	Referent	-1.5 (P=0.37)
**HDL Cholesterol**												
Entire Sample												
Unadjusted	Referent	-2.6 (P<0.01)	Referent	-0.26 (P=0.85)	Referent	-1.0 (P=0.14)	Referent	-2.5 (P<0.01)	Referent	1.3 (P=0.60)	Referent	0.24 (P=0.61)
Adjusted^b^	Referent	**-2.4 (P<0.01)**	Referent	-1.7 (P=0.25)	Referent	-1.2 (P=0.04)	Referent	**-3.2 (P<0.01)**	Referent	0.64 (P=0.76)	Referent	**-1.6 (P<0.01)**
Men												
Unadjusted	Referent	-1.4 (P=0.05)	Referent	2.0 (P=0.75)	Referent	-1.4 (P=0.01)	Referent	-2.1 (P=0.04)	Referent	0.02 (P=0.99)	Referent	-1.3 (P=0.01)
Adjusted^b^	Referent	**-1.8 (P=0.01)**	Referent	1.5 (P=0.81)	Referent	**-1.6 (P=0.01)**	Referent	-2.1 (P=0.05)	Referent	-0.33 (P=0.88)	Referent	**-1.4 (P=0.01)**
Women												
Unadjusted	Referent	-3.4 (P<0.01)	Referent	-2.1 (P=0.15)	Referent	-0.39 (P=0.80)	Referent	-3.7 (p<0.01)	Referent	4.5 (P=0.45)	Referent	-1.1 (P=0.10)
Adjusted^b^	Referent	**-3.1 (P<0.01)**	Referent	-1.9 (P=0.16)	Referent	-1.0 (P=0.50)	Referent	**-4.2 (p<0.01)**	Referent	4.6 (P=0.43)	Referent	-1.5 (P=0.04)
**Total/HDL Ratio**												
Entire Sample												
Unadjusted	Referent	0.26 (P<0.01)	Referent	0.13 (P=0.48)	Referent	0.26 (P<0.01)	Referent	0.29 (P<0.01)	Referent	-0.05 (P=0.86)	Referent	0.01 (P=0.93)
Adjusted^b^	Referent	**0.19 (P<0.01)**	Referent	0.10 (P=0.61)	Referent	**0.23 (P<0.01)**	Referent	**0.29 (P<0.01)**	Referent	-0.01 (P=0.98)	Referent	0.09 (P=0.06)
Men												
Unadjusted	Referent	0.27 (P<0.01)	Referent	0.04 (P=0.94)	Referent	0.31 (P<0.01)	Referent	0.32 (P<0.01)	Referent	-0.05 (P=0.87)	Referent	0.10 (P=0.20)
Adjusted^b^	Referent	**0.24 (P<0.01)**	Referent	-0.18 (P=0.78)	Referent	**0.26 (P<0.01)**	Referent	0.23 (P=0.07)	Referent	-0.04 (P=0.88)	Referent	0.09 (P=0.27)
Women												
Unadjusted	Referent	0.24 (P<0.01)	Referent	0.13 (P=0.41)	Referent	0.17 (P=0.09)	Referent	0.35 (P<0.01)	Referent	0.35 (P=0.28)	Referent	0.16 (P=0.01)
Adjusted^b^	Referent	**0.17 (P<0.01)**	Referent	0.10 (P=0.52)	Referent	0.17 (P=0.09)	Referent	**0.32 (P<0.01)**	Referent	0.41 (P=0.19)	Referent	0.11 (P=0.10)
**Triglycerides**												
Entire Sample												
Unadjusted	Referent	17.3 (P<0.01)	Referent	31.7 (P<0.01)	Referent	5.2 (P=0.57)	Referent	0.53 (P=0.97)	Referent	-58.3 (P=0.30)	Referent	22.4 (P<0.01)
Adjusted^b^	Referent	**15.1 (P<0.01)**	Referent	29.5 (P=0.45)	Referent	2.9 (P=0.75)	Referent	0.97 (P=0.94)	Referent	-58.8 (P=0.30)	Referent	**24.4 (P<0.01)**
Men												
Unadjusted	Referent	19.5 (P<0.01)	Referent	-7.3 (P=0.73)	Referent	9.3 (P=0.40)	Referent	-29.0 (P=0.41)	Referent	-59.5 (P=0.32)	Referent	34.0 (P=0.01)
Adjusted^b^	Referent	**19.8 (P<0.01)**	Referent	-20.0 (P=0.39)	Referent	5.8 (P=0.62)	Referent	-33.1 (P=0.33)	Referent	-61.4 (P=0.30)	Referent	32.0 (P=0.03)
Women												
Unadjusted	Referent	15.8 (P<0.01)	Referent	36.7 (P=0.39)	Referent	-3.2 (P=0.74)	Referent	23.0 (P<0.01)	Referent	34.5 (P=0.12)	Referent	23.8 (P<0.01)
Adjusted^b^	Referent	11.0 (P=0.04)	Referent	33.2 (P=0.40)	Referent	-2.9 (P=0.74)	Referent	**20.6 (P<0.01)**	Referent	23.4 (P=0.45)	Referent	**19.6 (P<0.01)**
**Atherogenic Index** ^c^												
Entire Sample												
Unadjusted	Referent	0.06 (P<0.01)	Referent	0.02 (P=0.71)	Referent	0.02 (P=0.30)	Referent	0.06 (P=0.009)	Referent	-0.13 (P=0.16)	Referent	0.04 (P=0.002)
Adjusted^b^	Referent	**0.05 (P<0.01)**	Referent	0.01 (P=0.84)	Referent	0.01 (P=0.39)	Referent	**0.06 (P<0.01)**	Referent	-0.13 (P=0.15)	Referent	**0.06 (P<0.001)**
Men												
Unadjusted	Referent	0.07 (P=0.002)	Referent	-0.04 (P=0.75)	Referent	0.04 (P=0.11)	Referent	0.02 (P=0.53)	Referent	-0.13 (P=0.19)	Referent	0.07 (P=0.002)
Adjusted^b^	Referent	**0.06 (P=0.002)**	Referent	-0.08 (P=0.59)	Referent	0.03 (P=0.23)	Referent	0.01 (P=0.65)	Referent	-0.13 (P=0.14)	Referent	**0.06 (P<0.01)**
Women												
Unadjusted	Referent	0.06 (P=0.002)	Referent	0.03 (P=0.63)	Referent	-0.01 (P=0.70)	Referent	0.10 (P<0.001)	Referent	0.10 (P=0.56)	Referent	0.07 (P=0.002)
Adjusted^b^	Referent	0.05 (P=0.02)	Referent	0.01 (P=0.76)	Referent	-0.01 (P=0.68)	Referent	**0.10 (P<0.001)**	Referent	0.10 (P=0.58)	Referent	**0.06 (P<0.01)**

Abbreviations: BMI, body mass index; CRP, C-reactive protein; HDL, high-density lipoprotein cholesterol; WC, waist circumference. ^a^Normal BMI (18.5-24.9 kg/m^2^); Overweight BMI (25.0-29.9 kg/m^2^); Obese BMI (30+ kg/m^2^); Normal WC (<102 cm for men and <88 cm for women); High WC (≥102 cm for men and ≥88 cm for women); Active (≥ 2000 MET-min-month); Inactive (<2000 MET-min-month). ^b^ ****Adjusted regression models controlled for *cholesterol medication use* (yes/no), *age* (years; continuous), *gender* (male/female)*, race-ethnicity* (Mexican American, non-Hispanic white, non-Hispanic black, and other), *mean arterial pressure* (mm Hg; continuous), *CRP* (mg/dL; continuous), *previous year changes in physical activity* (categorical; activity increased, decreased or stayed the same), *congestive heart failure* (yes/no), *coronary artery disease* (yes/no), *heart attack* (yes/no), *emphysema* (yes/no), *chronic bronchitis* (yes/no), *stroke* (yes/no), *diabetes* (yes/no) and *smoking status* (categorical; smokes every day, smokes some days, former smoker, never smoker). ^c^Atherogenic index (expressed in mmol/L) calculated as follows: (log_10_ (triglycerides/HDL-C). To convert HDL-C from mg/dL to mmol/L, divide by 38.67; to convert triglycerides from mg/dL to mmol/L, divide by 88.57. Bold values indicate statistical significance (*P*≤0.01) for the adjusted models. TC, triglycerides and HDL-C are expressed in mg/dL units.

## Discussion

High BMI and WC are independently associated with cholesterol profile,^5,6^ and PA is favorably associated with BMI, WC and cholesterol.^10^ However, the extent to which PA is associated with cholesterol concentrations across varying combinations of BMI and WC, is less clear. Thus, a novelty of this study is addressing the association between PA and cholesterol concentrations across various combinations of BMI and WC. The main findings for this study were that, after adjustments (including adjustment for age, gender, race-ethnicity and cholesterol medication use):

- Findings were not consistent across sex.
- There was little evidence to suggest a favorable association of PA on TC across varying BMI and WC combinations.
- There was sufficient evidence to suggest a favorable association of PA on HDL-C across varying BMI and WC combinations.
- Although not to the extent of HDL-C, there was some evidence to suggest a favorable association of PA on triglycerides and the atherogenic index across several BMI and WC combinations.
- When considering either sex, there was sufficient evidence to suggest a favorable association of PA on at least one of the evaluated cholesterol concentrations for each of the BMI/WC combinations with the exception of normal BMI and high WC.

Given that PA did not appear to have a beneficial or protective effect on TC for the different BMI/WC combinations, but did for HDL-C, the protective effects observed for the TC/HDL-C ratio is likely driven from the PA-HDL-C findings. These findings are in general agreement with other related research demonstrating stronger associations for PA and HDL-C when compared to TC.^24,25^ There is also other evidence demonstrating favorable effects of PA on triglyceride levels.^26^

Our non-consistent findings across sex are important to consider. Fewer studies on this topic have evaluated sex-specific associations regarding the relationship between PA and lipid profile. Using data from the Atherosclerosis Risk in Communities (ARIC) study, Monda et al^26^ also showed sex-varying associations. Other research has also demonstrated sex differences in the response of lipids to exercise,^27^ which is thought to be a result of menopausal status and use of hormone replacement therapy.^26^ At this point, explanation of such sex-specific lipid responses to exercise is not clear and worthy of future investigations. Another important consideration in this study is the notable observation that PA had a favorable association with at least one of the evaluated lipid concentrations across all BMI/WC combinations with the exception of those with a normal BMI but high WC. This observation may be a result of several factors observable from data in Table 1. Those in this group tended to have higher TC concentrations, were older and were predominately female (whom were less active). Additionally, recent work demonstrates that this group (normal BMI but high WC), compared to other BMI/WC combinations, has the highest mortality risk.^7^ Thus, PA may have less of an effect on this vulnerable at-risk group. Alternatively, this group may be less inclined to engage in PA.

Limitations of this study include the subjective assessment of PA and the cross-sectional study design, rendering causality not possible. Major strengths include the study’s novelty and national sample. The major finding of this study is that across most BMI and WC combinations, active individuals, compared to inactive individuals, have higher levels of HDL-C and lower triglyceride levels. Thus, these findings have several important clinical implications. These findings underscore the importance of clinician promotion of central adiposity control strategies such as directed PA considering that the beneficial effects of PA (regarding cholesterol) appears to be attenuated by central adiposity.

Additionally, given the varied cholesterol profile across BMI and WC combinations, when feasible, clinicians may wish to integrate WC measurements into their practice. Further, given the null associations between PA and those with normal weight central obesity, coupled with recent work suggesting that this group is at the highest mortality risk, clinicians may wish to identify and implement health-enhancing strategies among this vulnerable group.

## Ethical approval

The study procedures were approved by the ethics committee of the National Center for Health Statistics.

## Competing interests

None.

## Authors’ contributions

PDL and OA conceptualized the study and contributed to writing the manuscript. PDL conducted the analyses.
